# Vaccines for Mycoplasma Diseases of Small Ruminants: A Neglected Area of Research

**DOI:** 10.3390/pathogens11010075

**Published:** 2022-01-07

**Authors:** Katarzyna Dudek, Umit Sevimli, Sergio Migliore, Amirreza Jafarizadeh, Guido R. Loria, Robin A. J. Nicholas

**Affiliations:** 1 Department of Cattle and Sheep Diseases, National Veterinary Research Institute, 57 Partyzantów Avenue, 24100 Pulawy, Poland; katarzyna.dudek@piwet.pulawy.pl; 2 Pendik Veterinary Control Institute, OIE Reference Laboratory for CCPP, Pendik, Istanbul 34890, Turkey; umit.sevimli@outlook.com; 3 Area Diagnostica Specialistica, OIE Reference Laboratory for Contagious Agalactia, Istituto Zooprofilattico Sperimentale della Sicilia, Via G. Marinuzzi 3, 90129 Palermo, Italy; sergio.migliore@izssicilia.it (S.M.); guidoruggero.loria@izssicilia.it (G.R.L.); 4 Department of Microbiology, Faculty of Veterinary Sciences, Science and Research Branch, Islamic Azad University, Tehran 477893855, Iran; a.jafarizade@gmail.com; 5 The Oaks, Nutshell Lane, Farnham, Surrey GU9 0HG, UK

**Keywords:** small ruminants, mycoplasma, vaccines

## Abstract

Mycoplasmas cause some of the most economically important diseases of sheep and goats, including diseases listed by the World Organisation for Animal Health (OIE) such as contagious caprine pleuropneumonia (CCPP) and contagious agalactia (CA). Other important mycoplasma diseases include chronic respiratory and arthritic syndrome (CRAS) and atypical pneumonia, both present on all continents where small ruminants are farmed. Unfortunately, owing to a lack of investment, most commercial vaccines for these diseases are of poor quality, being mostly composed of killed bacteriocins of dubious or unknown efficacy. Several Mediterranean laboratories produce autogenous vaccines, but these can only be used on farms where outbreaks have been officially declared, and consequently have limited impact on disease nationally. Effective live vaccines are available, but their use is often restricted because of safety concerns. With the necessary safeguards in place, we argue for their greater use. This review examines reported vaccines for mycoplasma diseases of small ruminants and attempts to identify new candidate antigens that may enable the development of improved products. Vaccines for CCPP are covered elsewhere.

## 1. Introduction

Some of the most economically important diseases of sheep and goats are caused by mycoplasmas, small wall-less bacteria of the class *Mollicutes*. These include diseases listed by the World Organisation for Animal Health (OIE), such as contagious caprine pleuropneumonia (CCPP) and contagious agalactia (CA), as well as chronic respiratory and arthritic syndrome (CRAS) and atypical pneumonia, both present on all continents where small ruminants are farmed (Table 1). The true impact of these diseases is hard to determine, because their prevalence is mostly unknown as laboratories carrying out mycoplasma identification are not available everywhere. Furthermore, small ruminants are mostly managed by the poorer sector of the agricultural industry where the value of individual animals is low with very small or negligible profit margins. Consequently, the development of vaccines is a risky venture for commercial companies, which require large upfront expenditure with no certainty of uptake, particularly by subsistence farmers lacking the ability to pay for them. This lack of funding for effective vaccines is reflected in the poor quality of existing products available for the main mycoplasma diseases: nearly all of these are bacterins, some of which are of dubious efficacy. Even the most effective of the current vaccines may not provide immediate benefit, as it may take months before their value is seen. Compounding this, small ruminant producers can easily obtain over-the-counter antimicrobials and use these freely, often without veterinary supervision. This unregulated access and lack of veterinary advice means small ruminant producers are often unfamiliar with proper dosage, frequency and route of administration, length of therapy and withdrawal time of antimicrobials in animals intended for human consumption. More concerning, though, is the very real danger of antimicrobial resistance, an increasingly common problem seen in many animal mycoplasma diseases [1]. This is of more concern in cattle where there is widespread use of antimicrobials sometimes as growth promoters, a practice banned in many countries. While not a major problem yet in sheep and goats, some evidence of antimicrobial ineffectiveness is emerging in Europe in sheep flocks affected by CA [2].

This review focuses on research carried out for vaccines developed for CA, CRAS and atypical pneumonia. Vaccines for CCPP have recently been thoroughly reviewed [3,4], and so will not been included here.

## 2. Contagious Agalactia

CA is an economically important disease of sheep and goats with serious welfare effects. It is characterised by interstitial mastitis, arthritis, keratoconjunctivitis and, occasionally, abortion. Until recently, four mycoplasmas were associated with the disease primarily in goats: *M. agalactiae*, *M. mycoides* subsp. *capri*, *M. capricolum* subsp. *capricolum* and *M. putrefaciens.* However, recently, it was proposed that only one, *M. agalactiae*, should be considered the cause of classical CA [5]. The authors identified distinct differences in clinical presentation caused by the mycoplasmas and emphasised that only *M. agalactiae* is subject to animal disease regulations nationally and internationally. For these reasons, we will confine discussion of CA vaccines to those against *M. agalactiae*. Except for a few reports from Jordan and Iran, where sheep and goats are farmed together [6,7], only *M. agalactiae* is of concern to sheep farmers.

### 2.1. Inactivated Vaccines

The first attempts at producing CA vaccines were made in France in the 1920s, but were not greatly protective in animals [6]. Thirty years later, a live vaccine made from milk, brain and mammary gland homogenates from infected sheep was reported to be highly effective in flocks in Italy. This vaccine was in regular use until it was discontinued following severe outbreaks of scrapie in sheep and goats caused by the inadvertent inclusion of brain tissue containing scrapie prion protein [8]. Iran has a long history of producing CA vaccines, and showed very early on that a formalised vaccine adjuvanted with saponin was superior to both egg-cultivated mycoplasma inactivated with formalin and cell-culture-grown mycoplasmas adsorbed with aluminium hydroxide [6].

In Europe and western Asia, both commercial inactivated, adjuvanted vaccines and autogenous vaccines are available (Table 1), and are inactivated with formalin or phenol and adjuvanted with aluminium hydroxide, mineral oil, or saponin. Tola et al. [9] reported that vaccines inactivated with phenol or saponin gave better protection against experimental infections than did those inactivated with formalin, sodium hypochlorite or heat. However, to date, no single vaccine has been universally adopted and no standard methods of preparation and product evaluation have been applied. The strain, inactivation method and the adjuvant included in the vaccine are all key factors for its efficacy across strains [10]. Mineral-oil adjuvant-inactivated vaccines induce higher and longer-lasting protective immunity than the aluminium-hydroxide-absorbed vaccines, but they can also induce lesions at the injection site. Moreover, due to poor and/or transient immunogenicity, vaccination may need to be repeated at 4–6-monthly intervals. However, repeat and annual vaccination is often impractical and expensive, particularly for poorer farmers. Furthermore, their use, while alleviating clinical signs, does not always prevent shedding of *M. agalactiae* in milk or new infections. It is for these reasons that it is standard practice in southern Italy to combine vaccination with antimicrobial treatment (unpublished communication).

The limitations of these vaccines are best illustrated by a formalised vaccine which showed partial protection in goats challenged with *M. agalactiae*; however, it failed to prevent clinical signs following the introduction of naturally infected animals despite three vaccinations per year over six years [11]. Furthermore, in formalised vaccines, the shedding of *M. agalactiae* in the milk was not prevented [12].

In one of the few published comparative studies, Agnone et al. [13] tested four different vaccines, including a widely used commercial vaccine, using clinical signs and mycoplasma shedding as measures of protection in sheep. The results showed that the protection given by the vaccines varied considerably following contact challenge with experimentally infected ewes. A live attenuated vaccine conferred the best clinical protection, followed by a *M. agalactiae*-saponised vaccine. A commercial formalised vaccine was not protective at all against this disease. There is also concern in Greece that commercial CA vaccines may not be as effective in goats as they are in sheep [14]. It is possible that, in some cases, vaccine breakdown may be caused by mycoplasmas involved in CRAS [15]. To attempt to overcome this problem, a trivalent vaccine combining *M. agalactiae*, *M. mycoides* subsp. *capri*, and *M. capricolum* subsp. *capricolum* has been produced commercially, although there are no published reports of its efficacy (Table 2). Impractically, revaccination is recommended every 4–6 months.

An oil-emulsified inactivated vaccine against *M. agalactiae* showed promise in field trials in which sheep developed high antibody titres and were protected from infection by *M. agalactiae* with few clinical signs seen [16]. However, these vaccines are rarely commercialised, probably due to the cost of field trials and the complexities and vagaries of real infections in the field.

An early attempt to quality control inactivated CA vaccines in sheep was reported by Sarris [17], who described a scoring system made 14 days after subcutaneous challenge. The main criteria were the isolation of *M. agalactiae* from the udder, the main target organ, as well as body temperature and signs of mastitis. Sadly, no similar studies have been reported since. Of more concern is the lack of studies on cross protection of vaccine strains with heterologous strains.

### 2.2. Autogenous Vaccines

Following the ban on milk and brain vaccines, regulation of the manufacturing procedures of autogenous vaccines was increased and harmonised across the Istituti Zooprofilattici Sperimentali (IZS) in Italy. Their production is covered by the D.M. 17/3/94 No. 287, and is allowed only with specific authorisation from the Ministry of Health; working practices implemented in 2001 are detailed in the “Operating Guidelines for the Preparation of Stabulogenic Vaccines and Autovaccines”. In Sicily, an autogenous vaccine against CA is prepared following these guidelines. In brief, a *M. agalactiae* isolate is obtained from an outbreak, identified, cloned, and purified. The purified clone is quantified, inactivated, and checked for sterility before being emulsified with aluminium hydroxide. The IZS Sicilia uses formalin and, occasionally, saponin to inactivate isolates, while the IZS Sardinia prefers inactivating with phenol (Table 2).

A disadvantage of inactivated vaccines, unlike live ones, is that the mycoplasma content is massive, sometimes exceeding 10^10^ CFU/dose, significantly adding to the cost of production [10]. *M. agalactiae* is the main focus of vaccines, but can include *M. m. capri* and, less frequently, *M. c. capricolum* if the situation demands. Autogenous vaccines are a useful addition or practical alternatives to licensed products, but only bring about local solutions as their use is prescribed only in farms with confirmed outbreaks. Their effectiveness varies widely depending on many factors including the stage and severity of the outbreak when vaccination occurs. Little has been published on the use of autogenous vaccines in outside of Europe and western Asia.

### 2.3. Live Vaccines

Both live attenuated and inactivated vaccines for CA have been used many years in Turkey, with live vaccines being more protective of dams and their offspring [18]. The live vaccine was produced from the 40th passage of a local strain in selective media [19] but retained some virulence for lactating animals. The strain was further attenuated following another 30 passages, but this reduced protection in lactating females [19]. Consequently, this live vaccine was contra-indicated for lactating animals because of the risk that females may be transiently infectious [20].

In an in vivo study to assess the efficacy of two CA vaccines, a live attenuated vaccine used in the field for over 50 years in Turkey was compared with an experimental saponised-inactivated vaccine; the latter was adjuvanted with oil falba and contained the same strain as the live vaccine [21]. The inactivated vaccine provoked a strong humoral response as detected by ELISA, while the attenuated vaccine did not induce any antibody response. While 50% of unvaccinated controls showed CA disease signs, no clinical or pathological signs were seen in either group of vaccinated goats. The challenge strain was re-isolated from internal organs of all the controls and only a few goats in each vaccinated group. It was concluded that both vaccines could be used to control the spread of CA. The failure of the live vaccine to generate an IgG antibody response by ELISA to may be due to the random loss of virulence genes such as the NIF locus during attenuation [22]. Moreover, mutants lacking this locus do not elicit an IgG response in experimentally infected sheep, though it is not known whether the mutants were protective against challenge.

Live vaccines are relatively cheap, easy to administer, and could have a major effect in areas of endemic disease. However, as mentioned earlier, they may sometimes cause subclinical infection in lactating females [20], and are not recommended during this susceptible period. The live vaccine has been used for many years in Turkey, and evidence from the field suggests that it is highly protective with a long duration of immunity [21]. The live vaccine has also been shown to be effective in the face of an outbreak, with rapid reductions of clinical signs seen in affected animals, similar to those reported recently by Loria et al. [23] (see below).

### 2.4. Subunit Vaccine Candidates

Numerous new antigens have been identified that are immunogenic and, theoretically, could be used in new vaccines, but not many have so far made it into field trials. Indeed, few field trials have been carried out on existing inactivated vaccines, so the baseline from which future vaccines will be compared is not known. From the evidence to date, live vaccines probably offer the best opportunity for long lasting protection though the strains require manipulation to remove residual virulence in lactating animals. However, they can of course be used in younger animals, where this is not a problem. Meanwhile the impact of the wide genomic diversity and rapid switching of expression of the variable surface lipoproteins, Vpmas, of *M. agalactiae* strains on vaccine efficacy needs to be evaluated. Perhaps a cocktail of strains may be needed to protect against the numerous genotypes identified over the last decade [24,25].

All vaccine trials require the standardisation of the numerous animal infection models that have been described to date. These include challenge via the eyes, subcutaneously (SC), via the udder, orally, and by animal contact, with SC being reported to be the most successful in terms of disease and mycoplasma recovery [26]. In future, it may be possible that promising vaccine candidates can be screened using cell culture methods developed by Baranowski et al., rather than using animal models [22].

Much current research is focused on identifying immunogenic proteins that can be used in subunit vaccines. In addition, recent developments in epitope mapping, combined with whole genome sequence analysis, has facilitated the identification of immunogenic proteins that may be suitable candidates in vaccine development. Strongly immunogenic lipoproteins have been identified in *M. agalactiae*, as measured by reaction of antibodies in the sera of infected sheep. Originally identified in 2002 [27], the immunodominant adhesin P40 was shown to be expressed in all strains of *M. agalactiae* examined. More recently, it was shown to be recognised in infected animals and localised in the membrane [27], making it a strong potential vaccine candidate. Other proteins, including P48, or AvgC, may also be of value in future subunit vaccines [28].

The main target of these vaccines is often overlooked, as it is estimated that approximately 90% of cases affect the udder, with *M. agalactiae* entering via the teat canal. Consideration should be given to alternative routes of vaccine administration, such as to the organ via the intra-canalicular route.

### 2.5. Vaccine as a Treatment

Anecdotal evidence from the field has suggested that CA vaccines can bring about some improvement in the condition of affected sheep during outbreaks; indeed, in parts of southern Italy, this is normal practice [23]. In Central Sicily, a flock of sheep severely affected by CA, for which antibiotic treatment appeared ineffective, was given two doses of an inactivated vaccine against *M. agalactiae* two weeks apart. Mycoplasma shedding in the milk decreased over the next fortnight in nine of 11 ewes, selected for detailed examination, and was undetectable in all but one ewe two weeks after that. There was an improvement in milk quality and udder condition in nearly all the selected ewes. Similar findings have been reported for the live CA vaccine used in Turkey [21]. Confirmation of these findings is necessary before this use of CA vaccines to slow or prevent disease progression can become standard practice.

## 3. Caprine Respiratory and Articular Syndrome (CRAS)

*Mycoplasma mycoides* subsp. *capri* was merged and taxonomically superseded *M. m*. *mycoides* large colony (LC) because the two mycoplasmas are indistinguishable based on proteomic, genomic, and biochemical analyses [29]. This mycoplasma is the major pathogen associated with CRAS and has one of the widest geographic distributions of ruminant mycoplasmas, being found on all continents, including Australasia, where small ruminants are kept, and wherever CA and caprine pleuropneumonia are reported. However, the lack of awareness of mycoplasma diseases in many countries means that it is certainly under-reported. For a long time, *M. m. capri* was considered the cause of CCPP, and researchers were puzzled by their inability to isolate it from some affected goats, but very easily from others. However, then in 1976, MacOwen and Minette [30] isolated the highly fastidious *M. capricolum* subsp. *capripneumoniae* from a similar but pathologically distinct disease that affects only the lungs and pleural cavity. *M. m. capri* was shown to produce a much wider range of clinical signs, chiefly respiratory and arthritic disease, as well as septicaemia and occasionally mastitis, which was why it was formerly included in the group of causative mycoplasmas of CA [5].

The first attempts to produce a vaccine against diseases caused by *M. m. capri* were made at the Pendik Veterinary Institute in Turkey in the 1960s and led to the identification of the BQT strain, which after 38 passages in selective medium was protective in goats against challenge; other strains, including one that had been inactivated with formalin, did not protect [31]. This BQT vaccine is still in use today.

An Israeli vaccine comprised of *M. m. mycoides* LC (as it was called then), inactivated with formalin and emulsified with mineral oil and sorbitol, provided partial protection to 1-day-old and 6-week-old kids against a virulent challenge [32]. Unfortunately, the vaccine was never used widely, as the occurrence of the disease had fallen significantly over the following 20 years.

In India, the respiratory disease caused by *M. m. capri* was identified as a major impediment to goat farming [33], although no studies exist to show the prevalence of the disease sometimes wrongly termed CCPP. Attempts were made to develop a vaccine using a sonicated antigen adjuvanted with saponin. In trials, about 75% of vaccinated goats were protected against a virulent challenge for up to a year after vaccination [33]. Further experimental work was carried out comparing adjuvants with the sonicated antigen and found that a combination of saponin and aluminium hydroxide provided complete protection after one year, this despite a huge reported intra-tracheal challenge of 10^12^ CFU/goat [34]. Unfortunately, no large-scale trials have been conducted to evaluate this promising vaccine.

An increasing number of isolations of *M. m. capri* and, to a lesser extent, *M. c. capricolum* in Sardinia, where large numbers of goats are farmed, led to interest in vaccines for these mycoplasmas [35,36]. Autogenous vaccines were prepared and inactivated with phenol and successfully controlled outbreaks of CRAS when regularly used on selected farms.

Without data on the prevalence or economic impact of this mycoplasma in goats, it is hard to make a strong case for widespread vaccination with a monovalent vaccine other than as part of a vaccine for CA. Outbreaks can be explosive, as was seen in Sicily in two herds of goats where mortality rates in young kids reached 40% [37], but less virulent cases may also be common.

Complete attenuation of a *M. m*. *capri* strain using a synthetic genomics approach has already been achieved experimentally [3], and thus there will be substantial interest in further development of this approach. It is, however, unlikely to be incorporated into a CA vaccine until prevalence data justify its inclusion.

Although infections with *M. c. capricolum* may be severe, its prevalence is very low and, as might be expected, little or no work other than that carried out in Sardinia [35] has been carried out on vaccine development.

A multivalent formalin-inactivated vaccine incorporating all four mycoplasmas associated with CA appeared beneficial in field trials [38]; it was concluded, however, that a vaccine containing only *M. agalactiae* and *M. m. capri* would be adequate because of the infrequency of isolation of *M. c. capricolum* and *M. putrefaciens*. However, a commercial vaccine made in Italy incorporates *M. c. capricolum* with the two main pathogens, although it is hard to see what benefit it could provide.

Finally, the least worthy candidate for vaccine production is *M. putrefaciens*, a very infrequent isolate from goats with questionable pathogenicity; it is often isolated from healthy goats, but has been found occasionally in goat herds presenting agalactia, where it probably plays little role in disease progression [5].

## 4. Atypical Pneumonia

*Mycoplasma ovipneumoniae* is a causative and under-reported agent of chronic non-progressive (atypical) pneumonia (AP) in sheep and goats, affecting all ages and responsible for undetermined economic losses worldwide, often in combination with other respiratory pathogens [39,40,41]. Infection with *M. ovipneumoniae* can result in ciliostasis within the respiratory tract, often leading to polymicrobial pneumonia involving bacteria from the *Pasteurellaceae* family [42]. In the last decade, the mycoplasma has been shown to affect wild animals such as bighorn sheep (*Ovis canadensis*) and the Norwegian musk ox (*Ovibos moschatus*), from which the source of the disease outbreaks is invariably domestic small ruminants [43,44].

Several factors complicate the search for an effective vaccine for AP. These include a high strain variation in *M. ovipneumoniae* within and between domestic sheep flocks [45]. Evidence suggests immunity to *M. ovipneumoniae* is strain-specific [44]. The recently discovered intracellular nature of the pathogen being found within macrophages and neutrophils in the lung tissue of sick animals may also adversely affect immunity [43].

### 4.1. Inactivated Vaccines

The humoral response to *M. ovipneumoniae* is not always strongly expressed by the host following vaccination compared to natural infection, although further studies are required to confirm this; this may lead to further difficulties to develop effective vaccines [46,47,48]. Control is also hampered by the continuing lack of diagnostic tools enabling full identification of this fastidious and slow growing mycoplasma [46].

Humoral and cellular responses to a formalin-inactivated vaccine containing four *M. ovipneumoniae* isolates from two phylogenetically different branches of the 16S DNA tree were examined by Einarsdottir et al. [48]. Vaccinated sheep developed moderate humoral responses that were not boosted by an additional four doses of vaccine. Differences between the vaccinated and mock-vaccinated sheep were statistically significant, but there was a suspicion that the vaccinated group had been previously exposed casting doubt on the study. There was also limited proliferation of peripheral blood mononuclear cells (PBMCs) cultured with *M. ovipneumoniae* isolates post vaccination. Similarly, no significant differences in the number of PBMCs, as well as CD4 and CD8 T cells, were shown upon in vitro exposure to live *M. ovipneumoniae* compared to culturing with the heat-killed bacteria [48].

In a study to test the safety of a live and inactivated *M. ovipneumoniae* vaccine in domestic sheep, three vaccines were developed: one live and two bacterins containing different whole cell protein concentrations emulsified with Freund’s incomplete adjuvant [47]. No antibody responses were detected in animals inoculated twice with live or inactivated vaccine containing 50 µg cell protein. However, ewes immunised with 250 µg inactivated vaccine became strongly seropositive and showed significant serum inhibition activity to *M ovipneumoniae*; furthermore, these responses were transferred to their progeny. Adverse reactions were confined to the oil adjuvanted vaccines but were generally mild. This experiment provided evidence that vaccination could be applied to sheep with AP although the challenge and vaccine strains were the same. Further work on heterologous strains is needed.

### 4.2. Subunit Vaccine Candidates

Until recently there was little information on the antigenic and phase variations of *M. ovipneumoniae*, which is crucial for vaccine development. However, now studies show very high variability among *M. ovipneumoniae* isolates. In one study, DNA analysis revealed that of 43 sheep *M. ovipneumoniae* field isolates tested, over 40 different profiles were seen distributed between six and ten similarity clusters [45]. Additional analysis using pulse field gel electrophoresis with the restriction endonuclease *Sma*I revealed forty profiles within four similarity clusters. The high genetic heterogeneity of the strains studied was confirmed by the highly variable protein expression shown by SDS-PAGE and Western blotting [49]. This very high degree of variability among tested isolates, especially within the same individual and flock, is believed to be the cause of severe outbreaks, and significantly complicates vaccine development [45,49].

Furthermore, a study of the phenotypic and genomic analysis of total of 23 caprine and ovine field *M. ovipneumoniae* strains revealed a high degree of heterogeneity both within and between herds [49]. This study, regardless of the methods used, found differences between goat and sheep strains, which in most cases was reflected in the strains belonging to different clusters. Most of the thirty identified proteins were immunogenic, four of which, with molecular masses of 36, 38, 40 and 70 (±3) kDa, were considered predominant in both sheep and goats, and were selected as the potential vaccine candidates [47].

A comparison of seven housekeeping genes also showed high genetic variability among 34 ovine *M. ovipneumoniae* isolates in Iceland [48]. However, elongation factor Tu (EF-Tu), one of these housekeeping genes, showed no amino acid sequence variation among isolates studied and was identified as a possible candidate for a recombinant *M. ovipneumoniae* vaccine [48]. Heat shock protein 70 (HSP70), the membrane-associated protein of *M. ovipneumoniae*, has also been identified as highly immunogenic in in vivo mice studies and proposed as a potential protective antigen for subunit vaccine development [50]. In mice immunised with recombinant EF-Tu or HSP70, strong humoral responses were observed, expressed by higher serum specific IgG titers particularly with HSP70. This was reflected in significant stimulation of IgG1 and IgG2a, indicating both proteins induced a mixed Th1/Th2 response. Analysis of serum cytokine concentration also showed a clear stimulation of both Th1/Th2 cytokines in the immunised mice, again more strongly reported for rHSP70 protein. Both recombinant proteins seem to be capable of inducing a strong innate response. The results confirm the potential of rHSP70 protein as a Th1 cytokine-like adjuvant for the development of *M. ovipneumoniae* vaccines. Additional confirmation of the suitability of these two proteins was their stimulation of greatly increased amounts of IFN-γ^+^ secreting spleen lymphocytes. Moreover, antisera of mice immunised with rEF-Tu or rHSP70 were able to inhibit the growth of *M. ovipneumoniae* in vitro. Generally, rHSP70 performed better of the two proteins and certainly superior to *M. ovipneumoniae* extracts [48]. The immunogenic properties of the HSP70 protein had previously been shown in a study using the convalescent sera derived from the *M. ovipneumoniae*-infected sheep. This showed the presence of the recombinant C-terminal portion of the HSP70 protein in the positive sera, indicating a significant role for this protein in inducing an immune response in the course of infections with this mycoplasma. As in the study of Jiang et al. [50], the high potential of this protein as a vaccine antigen or adjuvant in the control of infections with *M. ovipneumoniae* was also indicated here [51].

## 5. Conclusions

Vaccination of small ruminants for mycoplasma diseases leaves a lot to be desired. Most vaccines used today are formalin-inactivated whole-cell vaccines, containing mycoplasmas with limited or no published efficacy. Indeed, one inactivated CA vaccine could not protect sheep from natural challenge despite annual vaccinations over the previous three years [11]. Furthermore, in a small trial, no potency was evident after vaccination using a commercial vaccine followed by contact challenge [13]. Interestingly, a live attenuated vaccine, successfully used in Turkey for many decades, was safe and protective [21]. Another advantage is that this live vaccine does not provoke an antibody response as detected by ELISA, so vaccine antibodies could be distinguished from natural infection [21]. While larger trials on commercial products are necessary, consideration should be given to using live vaccines, which are currently not allowed in Europe, in endemically affected areas.

Lung lesions caused by *M. ovipneumoniae* are commonly seen in abattoirs worldwide, although they are rarely reported officially [52]. Furthermore, the withdrawal of the Pasteurella vaccine in New Zealand because of its ineffectiveness against ovine respiratory disease suggested that *M. ovipneumoniae* was having a real impact there [52]. Its implication in disease in bighorn sheep and musk ox, which exist in small numbers, indicates its seriousness, and attempts at vaccination are underway, but need to be accelerated. Ideally, a vaccine incorporating bacterial pathogens like *Mannheimia haemolytica* and, as yet, undetermined numbers of representative strains of *M. ovipneumoniae* would be most likely to benefit the sheep industry.

The development of DNA vaccines and recombinant vaccines has been reported for many mycoplasmas of livestock, but few have yet been tried in the field. Even if successful in the short term, they are unlikely to provide long-term protection, and will probably require multiple doses. Sadly, based on developments in other areas of animal disease, there is little evidence that these innovative products will greatly improve the health of small ruminants affected by mycoplasma diseases [14]. Consequently, the prohibition of live vaccines is illogical and obsolete, as there are many live and recombinant vaccines available today against many pathogens of livestock such equine influenza, West Nile disease, Aujeszky’s disease, classic swine fever, bovine herpesvirus, swine rotavirosis, bovine mucosal disease (BVD/MD), bovine syncytial virus diseases, and others that affect domestic species even more widespread in the EU. Moreover, the recent introduction in Europe of Reg. CE 2016/429, which delists CA from the notifiable diseases, should allow access to registered products like live vaccines through veterinary prescription [14]. Of course, safety checks and careful monitoring of the spread of live vaccine strains to wild animals and unvaccinated flocks are required. In conclusion, it is time to use more effective prophylaxis, particularly for mycoplasma diseases like CA, for which inactivated vaccines remain suboptimal.

## Figures and Tables

**Table 1:** Characteristics of the most commonly isolated mycoplasmas of small ruminants.

Mycoplasma Species	Disease ^1^	Distribution	Disease Severity ^2^	Vaccine Available
*M. c. capripneumoniae*	CCPP	Africa, Western Asia	+++	Inactivated
*M. agalactiae*	CA	Mediterranean, Western Asia	+++	Inactivated, live
*M. m. capri*	CRAS	worldwide	++	Component of CA vaccines, Experimental
*M. c. capricolum*	CRAS	infrequent	++	Component of CA vaccines
*M. putrefaciens*	Agalactia	infrequent	+/−	None
*M. ovipneumoniae*	AP	worldwide	+	None, experimental
*M. arginini*	none proven	worldwide	−	None
*M. bovigenitalium ^3^*	infertility	worldwide	+/−	None
*M. conjunct* *ivae*	IK	worldwide	++	None

^1^ CCPP contagious caprine pleuropneumonia; CA contagious agalactia; CRAS chronic respiratory and arthritis syndrome; AP atypical pneumonia; IKC infectious keratoconjunctivitis. ^2^ +++ severe; ++ moderate; + mild; +/− variable. ^3^ formerly M. ovine/caprine serogroup 11.

**Table 2:** Characteristics of a selection of currently available vaccines for contagious agalactia in Europe and western Asia.

Name	Manufacturer	Country	Type	Spp/Strain	Dosage
Algontex	CZ Veterinaria S.A.	Spain	inactivated	*M. agalactiae* N262	5 × 10^8^ CFU/dose 2 doses then every 6 months
Ovax agalassia	Fatro S.p.A.	Italy	inactivated	*M. agalactiae*	10^9^ CFU/mL
Aglovax	MSD Animal Health S.r.l.	Italy	inactivated	*M. agalactiae*	10^10^ CFU/mL
				*M. m. capri*	10^10^ CFU/mL
				*M. c. capricolum*	10^10^ CFU/mL
Agalaxipra	Laboratorios HIPRA, S.A.	Spain	inactivated	*M. agalactiae* 784	not specified on data sheet
Myo-galax	Laboratorios Ovejero S.A.	Spain	inactivated	*M. agalactiae*	not specified on data sheet
Pulmovac ^1^/ Capridoll ^1^	Vetal/ Dolivet	Turkey	live	*M. m. capri* BQT	10^6^ CFU/mL
Mycolaxi ^1^/ Laxydoll ^1^	Vetal/ Dolivet	Turkey	live	*M. agalactiae* AIK 40p	2.5–3 × 10^5^ CFU/mL
Agalactivac oil ^1^/ Laxydoll oil ^1^	Vetal/ Dolivet	Turkey	inactivated	*M. agalactiae* AIK	2 mg protein/mL
Agalactia Vac	Razi Vaccine & Serum Institute	Iran	inactivated	*M. agalactiae*	10^9^ CFU/mL
Agalactia ^2^	IZSs	Italy	inactivated	*M. agalactiae*	10^8^ CFU/mL

^1^ Vaccines previously made at the Pendik Veterinary Control Institute. ^2^ Autogenous vaccines produced by the Italian Istituti Zooprofilattici Sperimentali are regulated by D.M. 17/3/94 No. 287 under specific authorisation from the Ministry of Health. Vaccine may incorporate *M. m. capri* and/or *M. c. capricolum* and/or *M. putrefaciens*.

## Data Availability

Not applicable.
